# Phase-separating resins for light-based three-dimensional printing of oxide glasses

**DOI:** 10.1038/s41598-024-63069-w

**Published:** 2024-05-29

**Authors:** Lorenzo Barbera, Henry Korhonen, Kunal Masania, André R. Studart

**Affiliations:** 1 Complex Materials, Department of Materials, ETH Zürich, 8093 Zürich, Switzerland; 2 Present Address: Shaping Matter Lab, Faculty of Aerospace Engineering, Delft University of Technology, 2629 HS Delft, The Netherlands

**Keywords:** Materials science, Glasses

## Abstract

Silica-based glasses can be shaped into complex geometries using a variety of additive manufacturing technologies. While the three-dimensional printing of glasses opens unprecedented design opportunities, the development of up-scaled, reliable manufacturing processes is crucial for the broader dissemination of this technology. Here, we design and study phase-separating resins that enable light-based 3D printing of oxide glasses with high-aspect-ratio features and enhanced manufacturing yields. The effect of the resin composition on the microstructure, mechanical properties and delamination resistance of parts printed by digital light processing is investigated with the help of printing experiments, compression tests and electron microscopy analysis. The chemical composition and microstructure of the cured resins were found to strongly affect the stiffness, delamination resistance, and calcination behavior of printed parts. These findings provide useful guidelines to enhance the reliability and yield of the DLP printing process of multicomponent silica-based glasses.

## Introduction

The three-dimensional printing of oxide glasses represents a major technological breakthrough with potential applications in microfluidics, filtration, biosensing and catalysis^1–7^. By enabling the manufacturing of complex-shaped, designer glasses in a digitized and automated process, this technology provides opportunities to shape glasses in geometries that thus far had only been possible to achieve through the ingenious manual labor of craftsmen. Today, oxide glasses with complex three-dimensional designs can be printed using a range of different extrusion- or light-based additive manufacturing techniques. Silica-based glasses are printable either directly in the molten state^3,8^ or through room-temperature consolidation followed by calcination and sintering^2,4,9^. Complex-shaped objects can vary in size from millimeters to several centimeters, depending on the additive manufacturing technology employed. Moreover, oxide glasses with tailored nanoporosity and chemical composition can be produced by proper selection and design of the feedstock material, printing method and processing conditions^2^.

Typical feedstock for room-temperature printing of silica-based glasses consists of reactive resins or suspensions loaded with inorganic precursors in the form of molecules^2,6,9–12^ or particles^4,5,13–20^. Phase-separating resins comprising a photoreactive monomer mixture and metal alkoxide precursors are particularly attractive due to the possibility of printing glasses from multiple oxides with light-tunable nanoporosity below the resolution of the printer^2,21^. The nanoporosity is generated through the polymerization-induced phase separation of the resin into inorganic-rich and organic-rich domains, followed by thermal decomposition of the organic phase. The size of the nanopores can be controlled within the range 200–1000 nm by changing the light intensity during printing^2^. If desired, the nanoporous glass can also be sintered at temperatures around 1000 °C to create complex structures with dense glass walls. Importantly, the composition of the porous or dense multicomponent glasses obtained with this method can be deliberately tailored through the choice of the metal alkoxides in the feedstock resin.

Despite its unique capability to control the chemical composition and nanoporosity of silica-based glasses, phase-separating resins have not been optimized to overcome manufacturing challenges that reduce the yield and throughput of the process. The layer-by-layer nature of digital light processing (DLP) printing and the extensive shrinkage of the printed objects during calcination are common sources of defects, which increase the percentage of rejects of the manufacturing process. Defects often occur in the form of delamination and cracking between the print layers. Such cracks are usually caused by interlayer internal stresses, which originate from differential shrinkage within the microstructure during curing and calcination. In addition to these manufacturing issues, current resin formulations have not yet been optimized to print complex geometries with high aspect ratios. Achieving high-aspect-ratio features is crucial to widen the design space of the process, thus enabling the exploitation of this technology in a broader range of applications.

We develop and study resin formulations for the DLP printing of oxide glasses with high-aspect-ratio features and enhanced processing yield. The type and content of organic and inorganic precursors are correlated with the microstructure of the printed resins to identify composition and processing conditions leading to a high yield of defect-free objects after printing and heat treatment. First, we perform compression tests on printed samples and print model pillar arrays to study the effect of the monomer composition on the mechanical properties and aspect ratio of printed resins. Next, model parts printed from distinct resin formulations are compared in terms of microstructure and delamination resistance during printing and calcination. Finally, we evaluate the printing capabilities of an optimized resin formulation by fabricating three-dimensional objects with multicomponent glass compositions and complex geometries.

## Results and discussion

**Figure 1:**
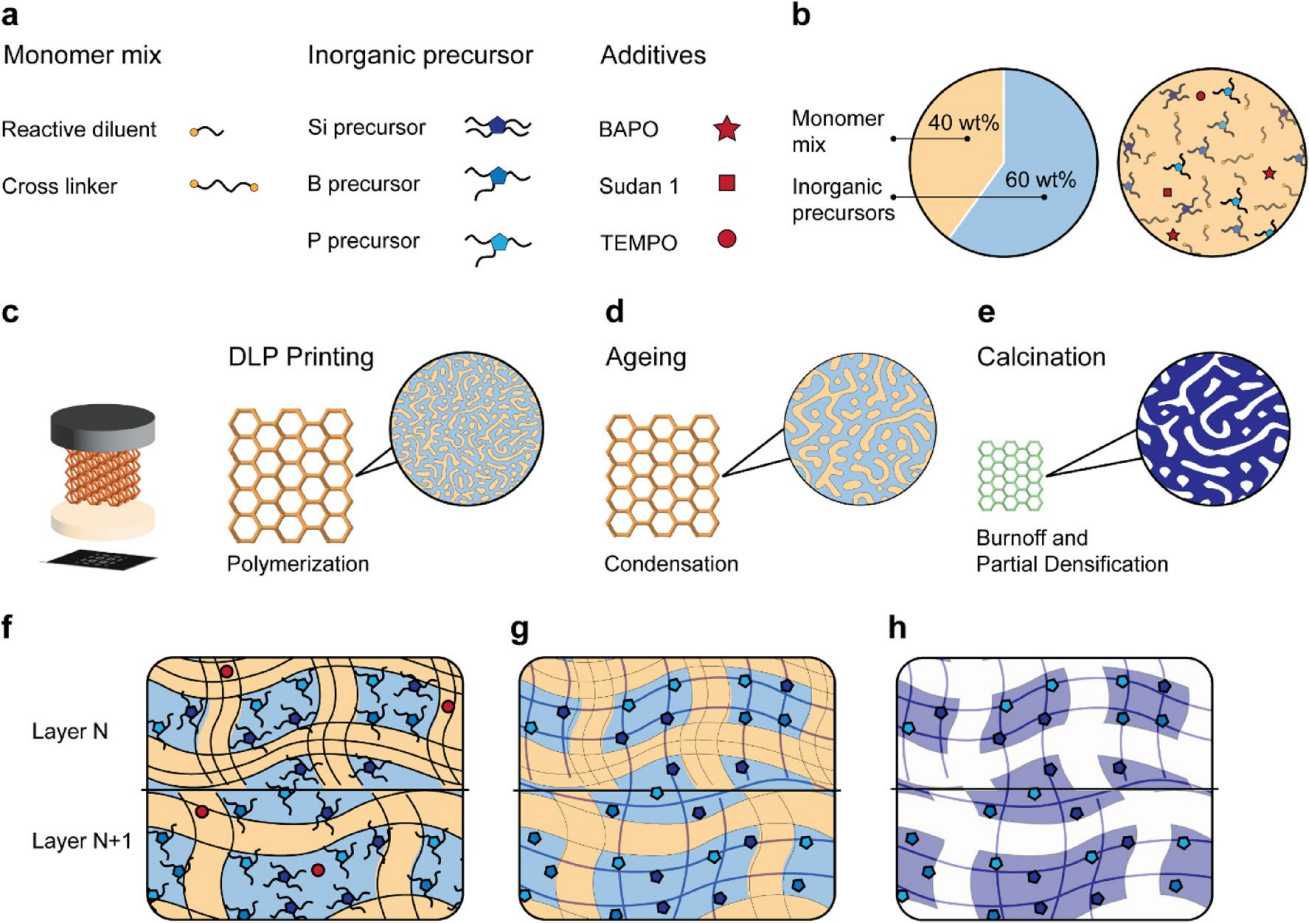
Manufacturing of multicomponent silica-based glasses by Digital Light Processing (DLP) 3D printing. (**a**,**b**) Monomer mixtures and inorganic precursors used in the hybrid resins formulated in this study. (**c**–**e**) Illustration of the main processing steps involved in the fabrication of oxide glasses using the DLP process. (**f**–**h**) Schematics of phase-separated microstructures expected after (**f**) printing, (**g**) ageing and (**h**) calcination.

Three-dimensional printing, aging, and calcination of hybrid resins are the three main steps that determine the aspect ratio, microstructure, and possible formation of defects during the manufacturing of complex three-dimensional glass objects using DLP technology (Fig. 1). In the printing step, a thin layer of resin (Fig. 1a,b) is illuminated below the build plate to allow for the photo-polymerization reaction and cross-linking of the organic monomers to take place (Fig. 1c). After polymerization, the part is detached from the resin-containing tray where a new thin layer of liquid resin is replenished. Although commercial implementations of the Digital Light Processing (DLP) technology differ in how the part is detached, the adhesion of the object to the build plate usually causes stresses within the polymerized layer, which might surpass the strength of the printed material and lead to undesired fracture. This effect is critical when printing small features with a high aspect ratio, since local stresses are amplified for such geometry. Therefore, the formulation of resins that can form stiff and strong polymerized layers is crucial to improve the achievable aspect ratios of complex printed objects.

In addition to solidification of the liquid resin, the light-induced polymerization process can induce phase separation of the organic and inorganic building blocks of the resin, leading to the formation of a three-dimensional network of interpenetrating domains (Fig. 1c). Because the polymerization reaction is faster under higher light intensity, light can be used to arrest the phase separation phenomenon at different timescales and thereby change the characteristic length scale of the interpenetrating domains. Aging of the polymerized part after printing may also lead to coarsening of the phase-separated domains along with condensation of the inorganic precursors (Fig. 1d). Such phase-separated microstructure can be converted into dense or porous glass after calcination above 600 ^∘^C (Fig. 1e). While the light intensity and the aging effects enable unique control over the microstructure of the printed resin, they may also introduce structural gradients within each individual print layer. Such gradients in domain size result from the decay of light intensity along the depth of the thin resin layer.

Layer-by-layer printing using phase-separating resins can therefore lead to intralayer microstructures, featuring interpenetrating domains with sizes that change periodically along the depth of the printed object (Fig. 1f–h). This creates a sharp microstructural transition at the interface between print layers, which results in mechanical mismatch and internal stresses within the printed part. If too high, such mechanical stresses lead to fracture and delamination between printed layers, a typical failure mode of parts printed by DLP and SLA technologies. The calcination process usually amplifies these fracture processes, due to additional stresses arising from the removal of thermally decomposed polymer and the associated extensive shrinkage of the material. Understanding the effect of the resin composition on the microstructure and mechanical properties of printed and calcined objects is crucial to establish guidelines for the DLP printing of hybrid resins into three-dimensional oxide glasses with high-aspect-ratio features at high manufacturing yields.

**Figure 2:**
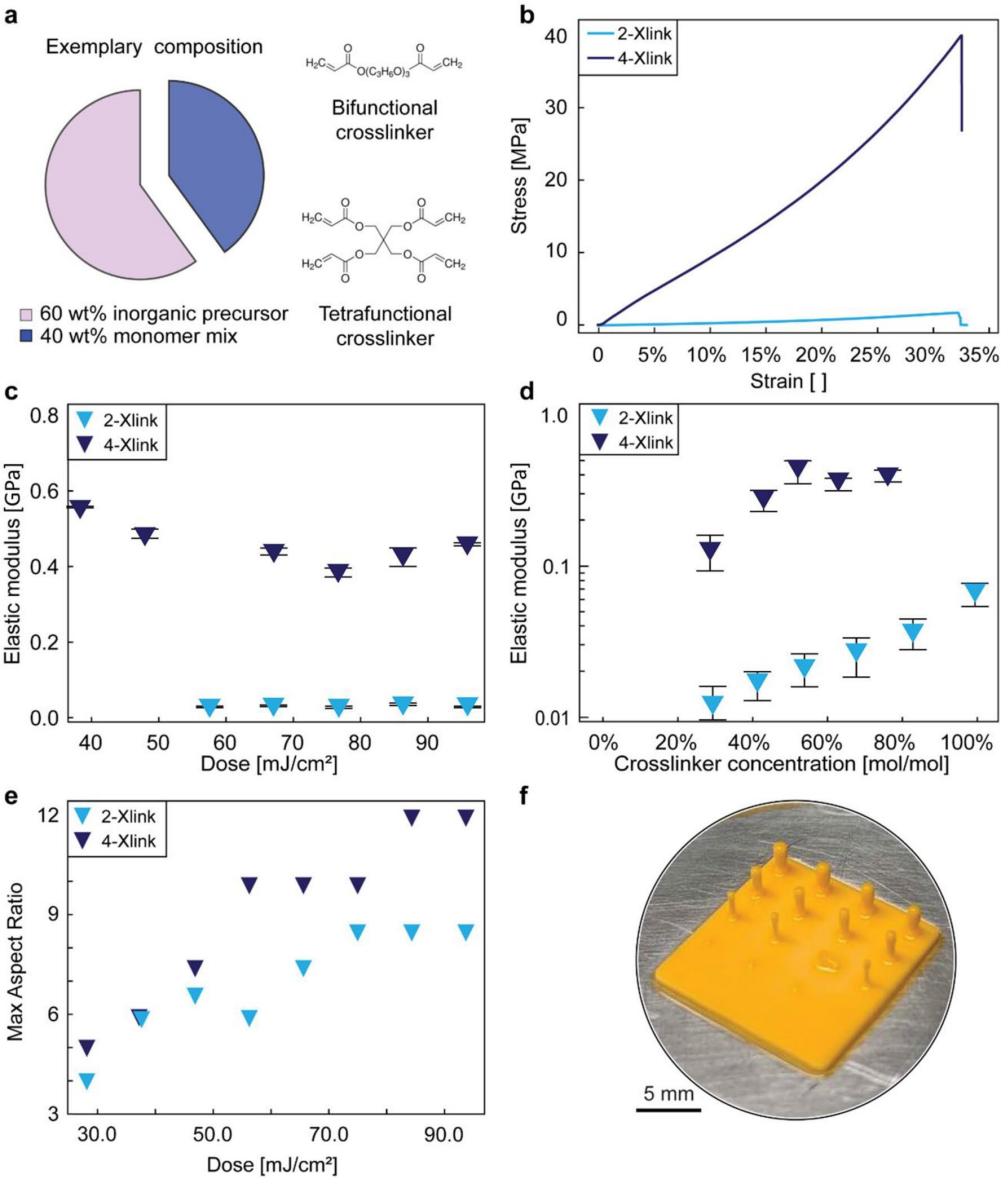
Mechanical properties and aspect ratio achieved with phase-separating resins containing distinct monomer mixtures. (**a**) Compositions of the two investigated resins, indicating the multifunctional monomers used to tune the cross-linking density and mechanical properties of the printed parts. (**b**) Effect of the resin composition on the stress–strain response of as-printed cylinders tested under uniaxial compression. (**c**,**d**) Elastic modulus of the as-printed cylinders as a function of (**c**) the light dose and (**d**) the crosslinker concentration for the two different formulations. The data points and error bars correspond to average values and standard deviations, respectively, taken from 5 different samples. (**e**) Maximum aspect ratio achieved by printing pillars with the bi-functional and tetra-functional crosslinker. (**f**) As-printed pillar arrays used to quantify the aspect ratios achievable with the two resin compositions.

To shed light on the role of the resin composition on the mechanical properties and aspect ratio of printed objects, we first designed two formulations with distinct monomer mixtures and fixed inorganic precursors. One monomer mixture comprises 2-[[(butylamino)carbonyl]oxy]ethyl acrylate (UA) and tripropylene glycol diacrylate (TPGDA) as monomer and bifunctional crosslinker, respectively, in a weight ratio of 2:8. This composition was developed in our earlier work ^2^ and is used here as a reference resin formulation, which we named 2-Xlink. To enhance the aspect ratio of printed parts, a resin containing 2-hydroxyethyl methacrylate (HEMA) and pentaerythritol tetraacrylate (PETA), a tetrafunctional acrylate crosslinker (4-Xlink), was evaluated relative to the base formulation. By increasing the functionality of the crosslinker from 2 to 4, we expect to form a polymerized network with higher crosslinking density and thereby improved mechanical properties. Although the viscosity of the tetrafunctional crosslinker (700 mPa.s at 25 ^∘^C) is higher than that of the bifunctional counterpart (57 mPa.s at 25 ^∘^C), the resin mixture containing PETA was found to be sufficiently fluid to be printed using DLP. The formulations are complemented with TPO as photoinitiator, Sudan I as light absorber, and a mixture of poly(diethoxysilane) (PDEOS), trimetyl borate (TMB) and triethyl phosphate (TEP) as inorganic precursors, resulting in a resin with 40 wt% monomer mixture and 60 wt% inorganic precursors (Fig. 2a).

The two resins were compared in terms of mechanical properties after polymerization by DLP-printing cylindrical samples with height of 8 mm and diameter of 4 mm and testing them under uniaxial compression. Although uniaxial compression does not represent the exact mechanical loading conditions experienced by the crosslinked resin during printing, we expect the shear-dominated fracture observed in the compression tests to be a good indicator of the resistance of the printed layer against the shearing forces developed in the printing process. The cylinders used for compression testing were printed with a light dose in the range 14–95 mJ/cm^2^ and an individual layer thickness set to 50 $\mu$m. Representative stress–strain curves and mechanical data obtained from the compression tests indicate that the resin with the tetrafunctional crosslinker (4-Xlink) reaches 20-fold higher elastic modulus and strength compared to the base formulation (2-Xlink, Fig. 2b). This improvement confirms our hypothesis that the density of cross-links directly affects the strength and stiffness of the polymerized resin. Moreover, the increase in mechanical properties was observed for the whole range of investigated light doses, suggesting that the polymerization reaction reaches full conversion for both resins (Fig. 2c).

To further evaluate the stiffness of the investigated resins, we also characterized samples with distinct concentrations of cross-linkers. In these tests, the content of inorganic precursors in the resin was fixed while the fraction of multifunctional cross-linker ranged between 6 and 40 wt% relative to the total resin composition. Our results show that the elastic modulus of specimens printed with 75 wt% of the tetra-functional crosslinker reaches a value of 0.43 GPa, which is sixfold higher than that of the stiffest formulation with the bifunctional monomer (Fig. 2d). Despite the high fraction of non-photocurable inorganic precursor (60wt%), the elastic modulus of the resin containing the tetra-functional crosslinker was found to be similar to those of commercial acrylate resins loaded with 20–30% of non-reactive organic phase^22^. Notably, the mechanical strength of the 4-Xlink formulation (40 MPa, Fig. 2b) is comparable to that of a pure acrylate resin used for light-based printing (48 MPa)^22^. With such improvement in mechanical properties, resins with the tetra-functional crosslinker enable printing of objects with higher aspect ratio compared to the reference formulation.

The larger aspect ratios achieved with the 4-Xlink formulation is demonstrated by printing arrays with multiple pillars with a constant height of 3 mm and diameters ranging from 50 to 1000 $\mu$m. To assess the success rate of the printing step we tested a total of 5 pillars for each of the 16 classes of specimens present in the array. The experiments reveal that pillars printed with the tetra-functional crosslinker can reach an aspect ratio that is up to 1.5 times higher than those obtained using the reference resin, considering a printing success rate of 80% (Fig. 2e,f). The maximum aspect ratio achieved with the tetra-functional crosslinker is 12, which corresponds to pillars with a height of 3 mm and a diameter of 250 $\mu$m. While our smallest printed diameter was 250 $\mu$m, we expect the minimum printable diameter to be given by the printer resolution (50 $\mu$m) if the aspect ratio is kept below 12. These findings illustrate the important role of multifunctional cross-linkers in increasing the mechanical properties and the aspect ratio of elements printed with hybrid resins.

**Figure 3:**
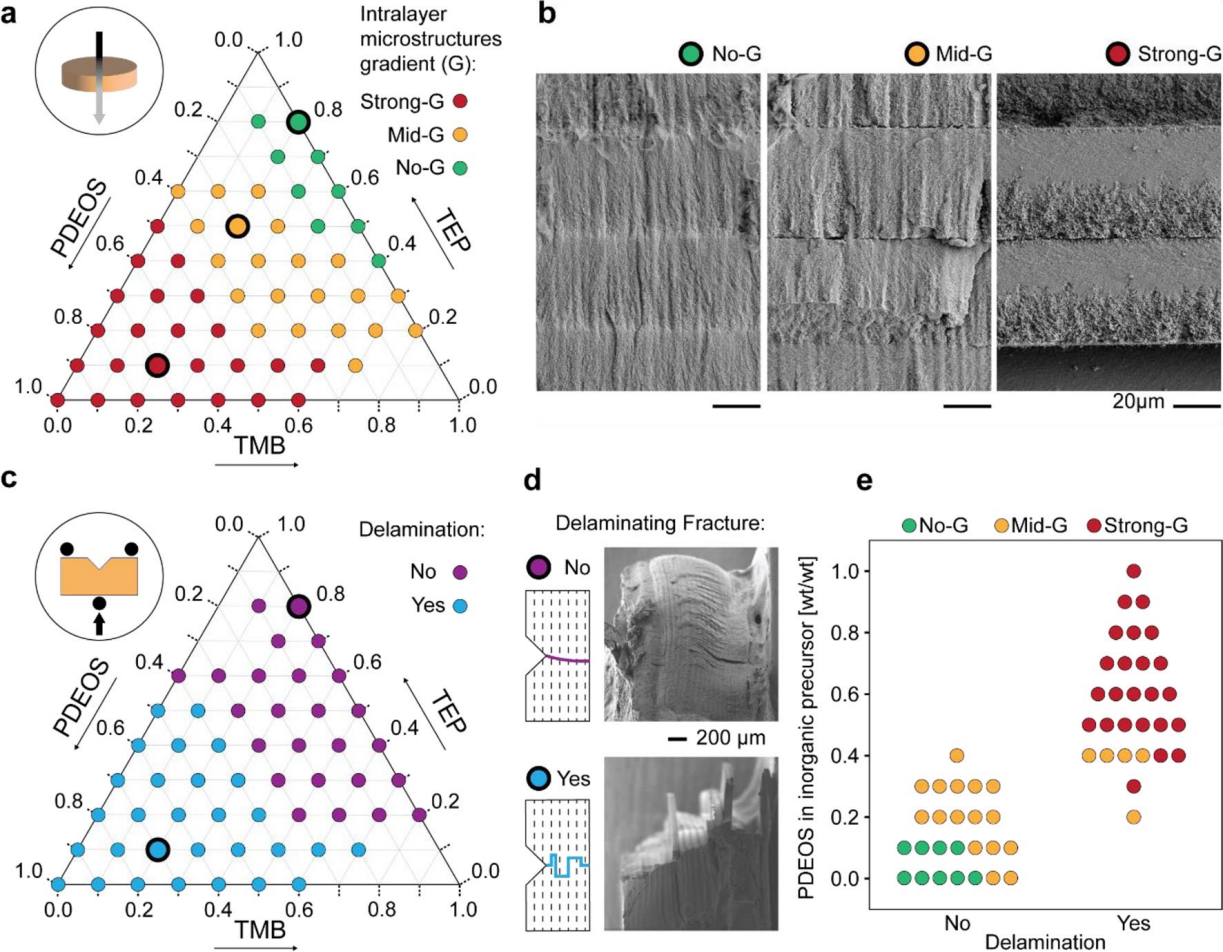
Phase-separation behavior, microstructure and delamination resistance of model parts printed from resins containing varying relative ratios of inorganic precursors indicating the observation of gradients (G) in the as-printed intralayer microstructure. (**a**,**c**) Ternary diagrams indicating (**a**) the optical appearance and (**c**) the delamination behavior of model objects printed from different resin formulations. (**b**) The microstructure of selected as-printed samples displaying non-graded (No-G), mildly graded (Mid-G) and strongly graded (Strong-G) intralayer morphologies. (**d**,**e**) Correlation between (**d**) microstructure and (**e**) delamination resistance of a total of 46 printed samples. The categorical plot (**e**) shows the number of printed parts that resist delamination depending on their intralayer morphology through the optical appearance proxy and the PDEOS content.

Hybrid resins with a high fraction of tetra-functional crosslinkers were further investigated by exploring the role of the inorganic phase on the phase separation process, the microstructure, and mechanical behavior of DLP-printed objects. In this experimental series, PDEOS, TMB, and TEP were used as inorganic precursors at different relative fractions while keeping a fixed total concentration of 60 wt% with respect to the entire formulation. By systematically varying the relative fraction of these precursors over a broad range, it was possible to investigate resins with distinct chemical compatibilities and therefore different responses to light-induced polymerization (Fig. 3).

The composition of the resin inorganic precursor was found to strongly influence the microstructure and the mechanical stability of model printed objects (Fig. 3a–e). To illustrate this effect, we first characterized the microstructure of printed discs by imaging cross-sections of polymerized samples in a scanning electron microscope (SEM). The images show the expected layered architecture and three typical intralayer microstructures depending on the resin composition (Fig. 3b): a non-graded layer (No-G), a mildly graded phase-separated layer (Mild-G), and a strongly graded phase-separated layer (Strong-G). Because they interact differently with light, these three microstructures can be easily identified by the optical appearance of the macroscopic printed discs. The non-graded samples remain transparent after printing, whereas the mildly and strongly graded discs turn, respectively, translucent and opaque upon polymerization of the resin. This simple visual feature was therefore used to infer the microstructure of the printed objects within the design space covered by our experiments (Fig. 3a).

For formulations with a fixed TMB content of 20 wt% with respect to the inorganic precursor composition, the layer’s microstructure changes from the non-graded to the mildly graded and strongly graded morphologies as the PDEOS concentration increases from 0 wt% to 70 wt% (Fig. 3b). On the basis of the microstructural gradients observed in our earlier work^2^, we infer that the intralayer gradients shown in the SEM images arise from the optical absorbance of the resin, which decreases the local light intensity and reduces the polymerization speed along the depth of the illuminated layer. As a result, a gradient in cross-linking density develops along the layer height, which in turn generates a stiffness gradient within the polymeric matrix. Such gradients cause earlier arresting of the phase separation process in the front, where the matrix is stiffer, compared to the back of the layer. This leads to a gradient in domain size of the interpenetrating polymeric and inorganic networks. Our experiments indicate that higher fractions of PDEOS amplifies this effect, favoring the formation of stronger gradients. This is possibly due to the lower chemical compatibility of this inorganic precursor with the polymeric phase. The transparency and the absence of a visible gradient in resins with low PDEOS contents suggest that phase separation is inhibited in these formulations (Fig. 3a).

To correlate the resin formulation with the mechanical stability of the printed objects, we designed samples with a notched layer geometry that allows for a qualitative assessment of the fracture mode and the ease of delamination between layers (Fig. 3c,d). Upon manual fracture at the notch site, delamination-prone samples break through the propagation of cracks along the layered interface (Fig. 3c). The delamination resistance of DLP-printed objects was determined by performing fracture tests on samples prepared from the same formulations used to assess the microstructure of printed layers.

Fracture experiments on 46 different compositions reveal a correlation between the intralayer microstructure and the delamination resistance of the printed samples (Fig. 3e). All specimens with non-graded morphology were observed to develop desirable trans-layer cracks, whereas fracture by delamination occurs in all samples with a strongly graded microstructure (Fig. 3d). Objects with a mildly graded intralayer show an intermediate behavior, with approximately 76% of the specimens withstanding delamination (Fig. 3c,e). These results corroborate the hypothesis that the absence of strong intralayer gradients enhances the probability of printed objects to resist delamination after the printing step. Because formulations with high PDEOS concentrations lead to highly graded, phase-separated morphologies, they demonstrate lower survivability after calcination. The strong phase separation tendency of such formulations can be explained by the poor chemical compatibility between the hydrophobic monomer mixture and the hydrophilic silica building blocks generated upon hydrolysis of the PDEOS precursor. The need to minimize the PDEOS concentration in the formulation imposes a challenge, since silica-based precursors are essential for glass formation upon calcination (Table S1 and Fig. S1).

Given that silica is a strong glass former and is the major oxide in several oxide glass compositions, we investigated approaches to develop Si-rich formulations that show superior mechanical properties and do not result in highly graded layer morphologies. To ensure mechanical stability of printed parts and avoid excessive gradient formation, both the monomer mixture, and the inorganic precursor formulation were modified to increase the chemical affinity between the organic and inorganic building blocks. First, monomer mixtures with a stronger hydrophilic character were combined with a pre-hydrolyzed inorganic formulation to promote intermolecular electrostatic interactions that reduce the tendency of the mixture to phase separate into strongly graded morphologies. Such interactions are also expected to limit the loss of inorganic precursors at the ageing and calcination stages. Second, the low-molecular-weight alcohol introduced along with the pre-hydrolyzed formulation as a cosolvent, is expected to improve the miscibility of the resin constituents. Finally, TEP was removed from the formulation and TMB was replaced by triethyl borate (TEB) to circumvent the low sol–gel reactivity of the former and the high volatility and water sensitivity of the latter.

**Figure 4:**
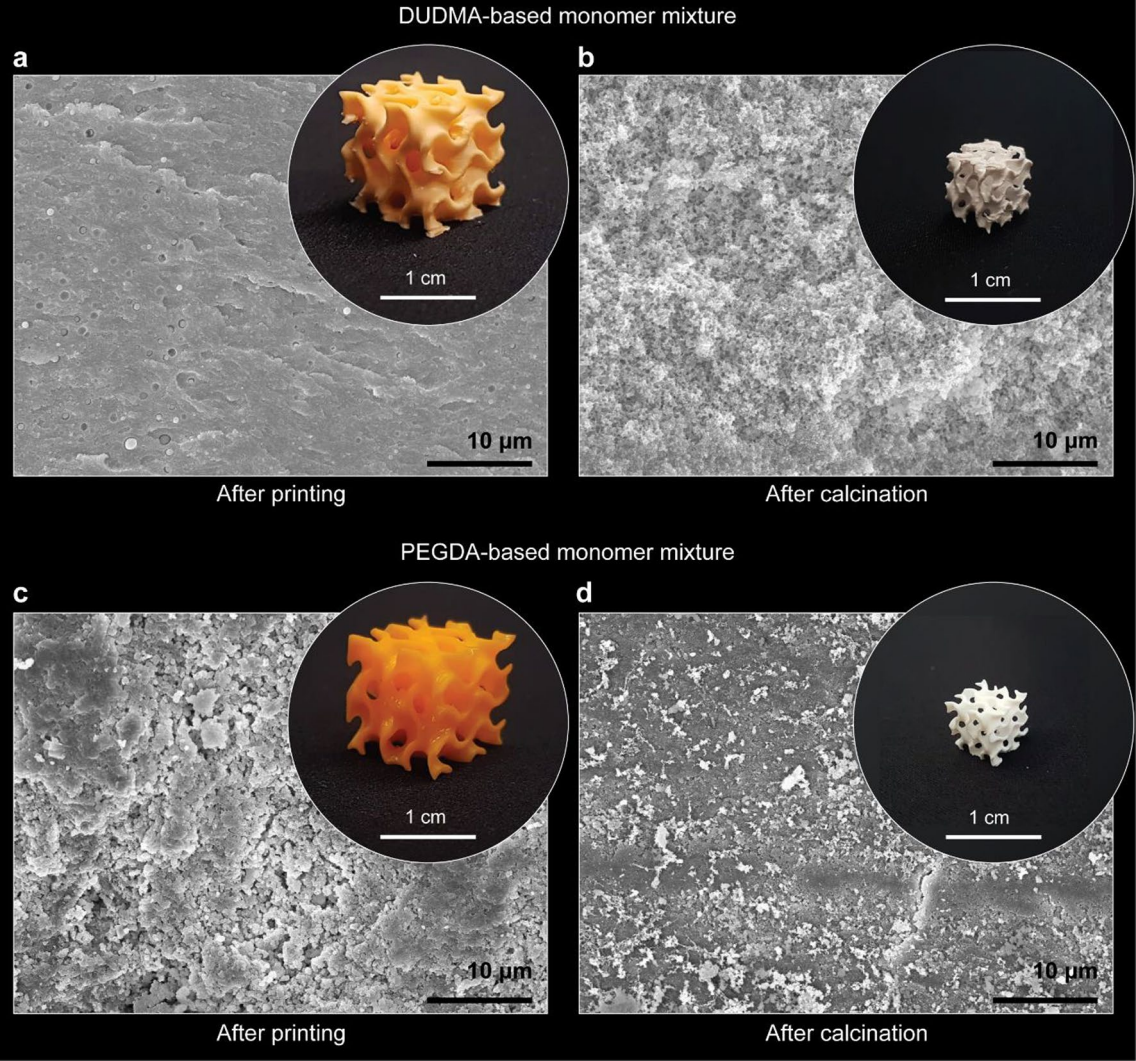
Gyroid-shaped glass structures fabricated by 3D printing and calcination of optimized resin formulations. (**a**–**d**) SEM images of non-graded microstructures (**a**,**c**) before and (**b**,**d**) after calcination of printed resins containing either (**a**,**b**) diurethane dimethacrylate (DUDMA) or (**c**,**d**) polytheylene glycol diacrylate (PEGDA) as hydrophilic monomers. The insets show photographs of the printed gyroid structures before and after calcination.

**Figure 5:**
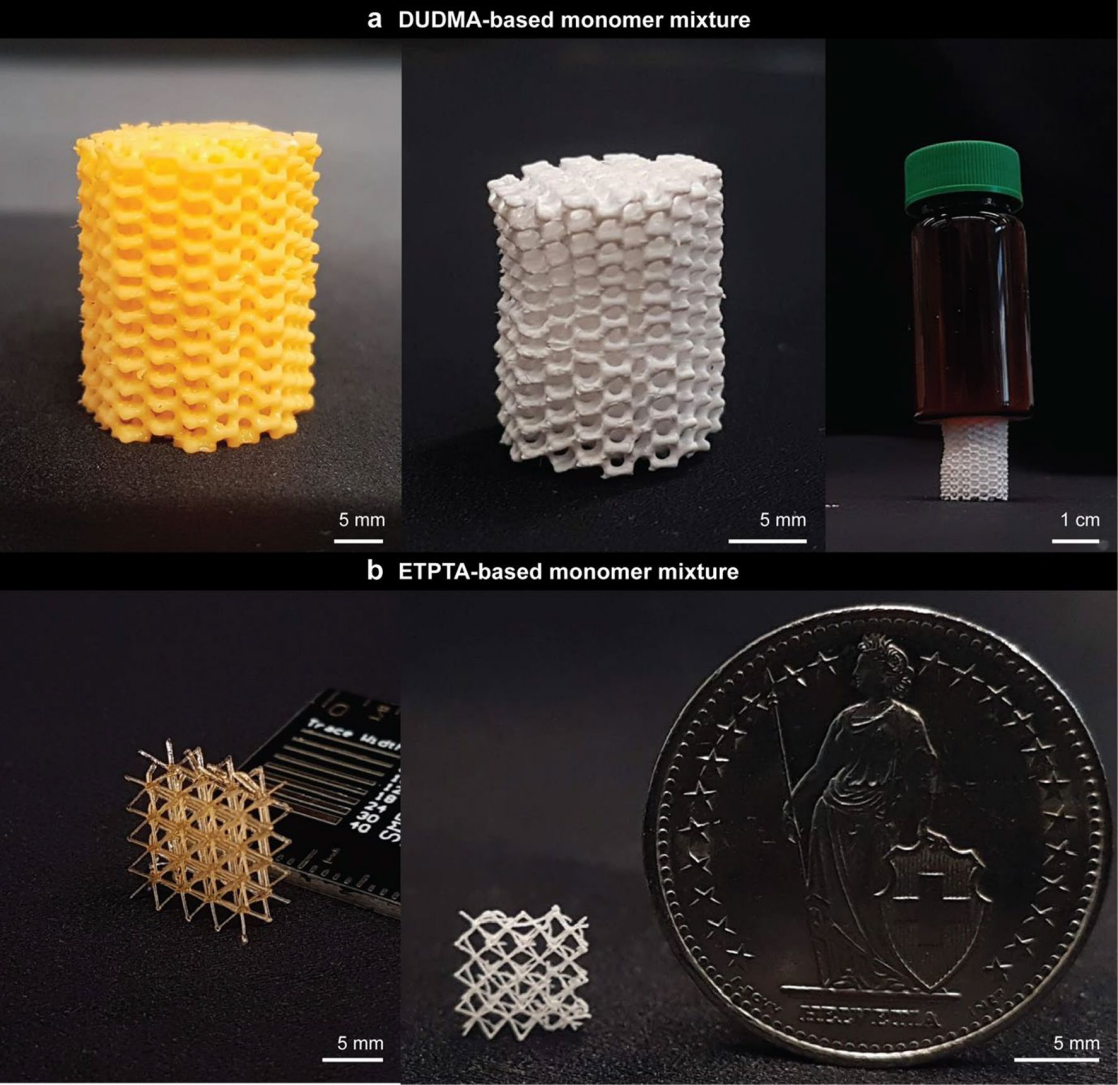
Silica-based glasses with complex architectures manufactured by printing and calcination of phase-separating resins. (**a**) Cellular structure printed from a resin containing diurethane dimethacrylate. The images show the part before (left) and after (right) calcination. The high mechanical stability of the calcined part is illustrated by placing a liquid-filled vial on top of the structure, (**b**) Printed (left) and calcined (right) lattice structures featuring struts with high aspect ratio.

Resins with optimized formulations could be successfully printed and calcined into crack-free, complex-shaped glass parts with porous microstructures (Figs. 4 and 5). To demonstrate this, we prepared resins with distinct compositions of the monomer mixture, which were designed to enhance the miscibility with the inorganic precursors. The inorganic precursor formulation comprising 80 wt% PDEOS and 20 wt% TEB, was combined in a 3:2 mass ratio with each one of the following hydrophilic crosslinkers: diurethane dimethacrylate (DUDMA), polyethylene glycol diacrylate (PEGDA, M_n_ = 250 g/mol), or ethoxylated trimethylolpropane triacrylate (ETPTA, M_n_ = 428 g/mol). Overall, the resin composition was as follows: 48 wt% PDEOS, 12 wt% TEB, and 40 wt% crosslinker. DUDMA is expected to interact favorably with the inorganic precursors via hydrogen bonds, whereas PEGDA and ETPTA show high chemical affinity towards the inorganic constituents through dipole–dipole van der Waal interactions of their ethoxylated chains. Scanning electron microscopy images of the polymerized resins before and after calcination confirm that the higher miscibility of the optimized resins results in non-graded morphologies (Fig. 4a,c).

Optimization of the resin formulation and its processing conditions opens the path to manufacturing of intricate geometries that cannot be printed using the initially reported formulation. We illustrate this by printing silica-based multicomponent glasses with exquisite architectures, including complex-shaped lattices with high-aspect-ratio struts (Fig. 5a,b). The non-graded intralayer microstructures of the as-polymerized resin are essential to produce such unique objects with high-aspect-ratio elements. By proper selection of the resin constituents, a broad range of oxide compositions can be covered to match the requirements of specific applications. Importantly, the reduced delamination and limited defects of the resin formulations developed in this study should lead to DLP processes that are sufficiently robust for up-scaled manufacturing of complex-shaped oxide glasses.

## Conclusions

The composition of phase-separating resins strongly affects the geometrical features, delamination resistance and calcination behavior of multicomponent oxide glasses printed using Digital Light Processing technology. The achievable aspect ratio of printed pillars can be improved 50% by utilizing tetra-functional monomers that increase the crosslinking density and mechanical properties of the cured resin. In terms of delamination resistance, we found that resin formulations that undergo mild phase separation are more likely to remain crack-free after the printing process, since they display a non-graded intralayer microstructure with reduced mechanical stresses between layers. The calcination of crack-free printed parts into complex-shaped structures requires optimized resins with high concentrations of silica inorganic precursors and low tendency to phase-separate into graded morphologies. Resins with high cross-linking density, high silica content and free from inhomogeneous intralayer microstructures enable the DLP-printing of complex-shaped multi-oxide glasses with aspect ratios up to 12. These capabilities demonstrate the potential of this glass printing technology to fulfill some of the specifications required for the up-scaled manufacturing of devices for microfluidic, sensing and catalytic applications.

## Materials and methods

### Materials

The chemicals 2-[[(butylamino)carbonyl]oxy]ethyl acrylate (urethane acrylate, UA) and tripropylene glycol diacrylate (bifunctional methacrylate crosslinker, 2-Xlink, TPGDA) were purchased from Merck KGaA, Darmstadt, Germany. 2-hydroxyethyl methacrylate (HEMA) was acquired from Thermo Fisher Scientific, Waltham, Massachusetts, USA. Pentaerythritol tetraacrylate (tetrafunctional acrylate crosslinker, 4-Xlink, PETA) was purchased from abcr, GmbH, Karlsruhe, Germany. Diphenyl(2,4,6-trimethylbenzoyl)phosphine oxide (initiator, TPO) and 1-phenylazo-2-naphthol (light absorber, Sudan I) were also obtained from Merck KGaA, Darmstadt, Germany. Poly(diethoxysilane) (45–47% SiO_2_, PDEOS) and trimethylborate (TMB) were purchased form abcr GmbH, Karlsruhe, Germany. Triethyl phosphate (TEP) was acquired from Merck KGaA, Darmstadt, Germany.

### Resin with bifunctional crosslinker

To obtain 10 g of resin with a bifunctional crosslinker (2-Xlink), chemicals were added to a 15 ml brown glass flask in the following order: TPGDA (3.20 g), UA (0.80 g), PDEOS (3.81 g), TEP (0.94 g), Sudan I (3.5 mg), TPO (100 mg), TMB (1.25 g). All liquid constituents were added directly into the flask with a 3 ml polyethylene pipette while measuring the mass using a laboratory scale (XS2 Dual Range, d = 0.01 mg, Mettler Toledo, Greifensee, Switzerland). Sudan I and TPO were weighed on a laboratory scale (XS4002S Delta Range, d = 0.01 g, Mettler Toledo, Greifensee, Switzerland) and added to the liquid. After adding TMB, the flask was closed tightly and sealed with parafilm, before subjected to hand shaking for 5 s and to sonication for 10 min to ensure complete mixing (Model 1510 Ultrasonic Cleaner, Branson Ultrasonics Corporation, Brookfield, Connecticut, USA).

### Resin with tetrafunctional crosslinker

To obtain 10 g of resin with a tetrafunctional crosslinker (4-Xlink), chemicals were added to a 15 ml brown glass flask in the following order: PETA (3.60 g), HEMA (0.40 g), PDEOS (3.81 g), TEP (0.94 g), Sudan I (3.5 mg), TPO (100 mg), TMB (1.25 g). The mixing protocol followed the procedure described above for the preparation of the formulation with the bifunctional crosslinker (2-Xlink).

### DLP printing

The printer used in this work is an Ember DLP 3D printer (Autodesk, San Francisco, California, USA) running on a modified firmware and resin vat. A custom-made vat holder was designed to hold a polystyrene Petri dish with a diameter of 5.5 cm, which was used as the resin vat. The bottom of the Petri dish was additionally coated with silicone (Silgard 184, Dow chemicals, Midland, Michigan, USA) and laminated with a fluorinated ethylene-propylene foil (McMaster-Carr Supply Company, Elmhurst, Illinois, USA) to minimize the adhesion between the dish and the printed part. Unless otherwise specified, all parts were printed with a layer thickness of 50 µm. Exposure times were adapted to obtain the best print quality based on the part’s geometry and size. The printer’s projector had an intensity of 19.2 mW/cm^2^.

### Compression testing

Mechanical testing was performed on two sets of samples prepared either from a specific resin exposed to different light doses or from resins with distinct crosslinker concentrations. FreeCAD was employed to design the samples while PrusaSlicer was used to slice the corresponding stl files. To minimize the printed contact area while maximizing the number of samples, five 2 mm × 8 mm (radius x height) cylinders were printed at the same time. Samples exposed to different light doses were prepared using a layer thickness of 50 µm and varying the exposure time between 0.75 s and 5 s, which corresponds to light doses in the range 14.4–95.9 mJ/cm^2^. Resins with different crosslinker concentrations were prepared by first mixing the inorganic precursors, the initiator, and the dye. In a second step, monomers were added according to their desired concentration. The container was then sealed and sonicated for 10 min. For each print, a layer thickness of 50 µm was used while the exposure time was kept constant at 3.0 s, which corresponds to a light dose of 57.5 mJ/cm^2^.

Printed parts were first carefully wiped dry with clean paper (Kimwipe) and cut off from the build head using a razor blade. All cylinders were stored in a glass Petri dish with a lid in order to minimize evaporation. Prior to testing, the mass, radius, and length of each specimen was measured. Compression measurements were carried out on an AGS-X (Shimadzu Corp., Kyoto, Japan) universal testing machine in compression mode. A 1 kN load cell (SSM-DAM-1000N) with 3 cm compression plate was driven at a constant speed of 0.5 mm per minute. All data was analyzed with a custom Python script.

### Microstructure-property diagram

The effect of the resin composition on the intralayer microstructure and on the delamination resistance of printed samples was investigated by preparing formulations with fixed monomer mixture and varying concentrations of inorganic precursors (Fig. 3, main text). Resins were mixed as follows: first, 3 g of each inorganic precursor composition were mixed, sonicated for 60 min, and stored for 1 day in a sealed glass vial to ensure complete mixing. On the following day, a stock solution comprising 10 wt% HEMA, 90 wt% PETA, 0.0875 wt% Sudan I, and 2.5 wt% TPO was prepared. After mixing, 2 g of the stock solution was added to each inorganic precursor composition and the resulting resin was again mixed. 1 mm-thick disks were printed with the Ember DLP printer using a 50 µm layer thickness and exposure time of 2.75 s. Immediately after printing, the disks were washed with isopropyl alcohol (IPA) and dried with a paper tissue. Next, the samples were inspected against a printed ‘ETH Zürich’ logo and assigned a class based on the appearance of the character ‘ü’. If the two dots above the character ‘ü’ were distinct, the resin was assigned the class ‘transparent’. If the dots were visible but not clearly distinguishable the samples was considered ‘translucent’. Otherwise, the disk was assigned to the class ‘opaque’. The inspection was conducted by naked eye under controlled illumination in a standard fume hood. The logo was printed with a commercial laser printer and the dots of the ‘ü’ character had a diameter of 263 µm and a center-to-center spacing of 327 µm.

### Delamination tests

For the delamination tests, two 2.5 × 3.0 x 7.0 mm prisms with a 1.25 mm-deep, 45° notch in the middle were printed using a layer thickness of 50 µm and exposure time of 2.75 s. After washing with IPA, the samples were dried and stored in a closed, unsealed box for 7 days to ensure condensation of the inorganic network. The samples were then fractured by hand in a 3-point-bending fashion and the fracture surface was inspected by eye. If delaminated layers are visible, the resin is assigned to the ‘delaminated’ class.

### Optimized resin formulations

Optimized resins were developed to print parts that resist delamination and can be converted into crack-free complex objects after calcination. This was achieved by formulating resins with high silica content and minimally graded microstructures after polymerization. The preparation of the resins involved the pre-hydrolysis of the inorganic precursors before mixing with the reactive monomers. The protocol followed for the hydrolysis of 100 g of inorganic precursor mix was as follows: 6.67 g of 10 mM aqueous solution of nitric acid (HNO_3_) were mixed with 10.0 g of ethanol and slowly added to a mixture composed of 66.7 g of PDEOS and 16.7 g of TEB. The mixture quickly turned milky thereafter. The solution was then kept closed and transferred to a stirring plate where it was mixed for 12 h before use. The pre-hydrolyzed solution was mixed with one of the following hydrophilic monomers to obtain the final resin: diurethane dimethacrylate (DUDMA), polyethylene glycol diacrylate (PEGDA, M_n_ = 250 g/mol), and trimethylolpropane ethoxylate triacrylate (ETPTA, M_n_ = 428 g/mol). The final resin contained 48 wt% pre-hydrolyzed PDEOS, 12 wt% pre-hydrolyzed TEB and 40 wt% monomer.

### Calcination of printed objects

Objects printed with the phase-separating resin were calcined in a furnace (Nabertherm LT, Germany) under atmospheric conditions with a heating rate of 0.4 °C/min. Isotherms at 250 °C for 6 h and at 700 °C for 4 h were performed to enable calcination of the organic phase and the generation of porosity in the final part.

### Supplementary Information

Supplementary Information.

## Data Availability

All data generated or analysed during this study are included in this published article and its supplementary information file.
