# Supplementary material for: Phase-separating resins for light-based three-dimensional printing of oxide glasses
Source: Sci Rep. 2024 May 29;14:12323. doi: 10.1038/s41598-024-63069-w (PMC11137103; doi:10.1038/s41598-024-63069-w)
Supplement: Supplementary file 1 — Supplementary Information. [file 41598_2024_63069_MOESM1_ESM.docx]

**Supplementary Information: Phase-separating resins for light-based three-dimensional printing of oxide glasses**

Lorenzo Barbera ^1^, Henry Korhonen^1^, Kunal Masania ^1,2^, André R. Studart ^1🞸^

^1^ Complex Materials, Department of Materials, ETH Zürich, 8093 Zürich, Switzerland

Current address:

^2^ Shaping Matter Lab, Faculty of Aerospace Engineering, Delft University of Technology, 2629 HS Delft, Netherlands

**Supplementary Information:**

The resin formulations used to study the effect of layer microstructure on the delamination resistance (Figure 3) were further investigated in terms of their calcination behavior (Table S1 and Figure S1). To this end, we DLP-printed pillars displaying each one of the three intralayer morphologies: non-graded, mildly graded and strongly graded. The distinct morphologies were obtained by varying the relative concentration of inorganic precursors, while keeping the fraction of the tetrafunctional monomer mixture constant at 40 wt% (Table S1). For calcination, the printed samples were slowly heated up to 650 °C following the standard protocol used to thermally decompose the polymer phase and to promote the condensation of the inorganic precursors into a mechanically stable percolating network.

***Table S1:*** *Concentrations of inorganic precursors in resins leading to distinct intralayer morphologies used for calcination experiments.*

| Class | PDEOS [wt%] | TMB [wt%] | TEP [wt%] |
| --- | --- | --- | --- |
| Non-graded | 0 | 12 | 48 |
| Mildly graded | 18 | 12 | 30 |
| Heavily graded | 42 | 12 | 6 |

To test the calcination behaviour of the resins, 9 cylinders, 8 mm tall and 4 mm in diameter, were printed using a layer thickness of 50 µm and and exposure time of 5.0 s, which corresponds to a dose per layer of 96 mJ/cm^2^. Following printing, the samples were aged for 9 days to ensure complete condensation in a closed but not sealed box. The calcination program consisted of a first ramp up to a temperature of 150 °C, which was held for 4 hours, followed by a second ramp to 650°C, where the samples were held for 4 additional hours. All heating rates were kept constant at 30°C/h.

Our experiments reveal that the printed specimens with a strongly graded morphology undergo severe delamination (Figure S1a), confirming our previous observations (Figure 3, main text). By contrast, no delamination is found in samples displaying the non-graded and mildly graded microstructures (Figure S1,b,c). In terms of calcination behavior, we observed extensive cracking and fragmentation of the non-graded and mildly graded samples, whereas the strongly graded part retained its overall shape after calcination. These results indicate that the success of the calcination step is not only dependent on the intralayer microstructure. Instead, we hypothesize that the presence of a high concentration of PDEOS is essential to preserve the mechanical integrity of the printed part during calcination.

The homogeneous and bright white coloration of the samples with high PDEOS concentration also suggest the formation of a silica-rich glass and complete removal of the organic phase during calcination of this resin. Conversely, the brownish color and lack of structural integrity of the non-graded and mildly graded specimens after calcination points towards an unstable inorganic network. This may be explained by the higher content of a phosphorous containing-precursor of these resins (Table S1), which shows high volatility [1] and low sol-gel reactivity [2]. Overall, our experiments indicate that a high concentration of silica precursor in the resin is essential for the successful calcination of the printed parts. Based on this finding, the resin was further optimized to ensure both high silica concentration and non-graded or mildly graded morphologies by designing the monomer mixture to be chemically more compatible with the PDEOS precursor (see main text).


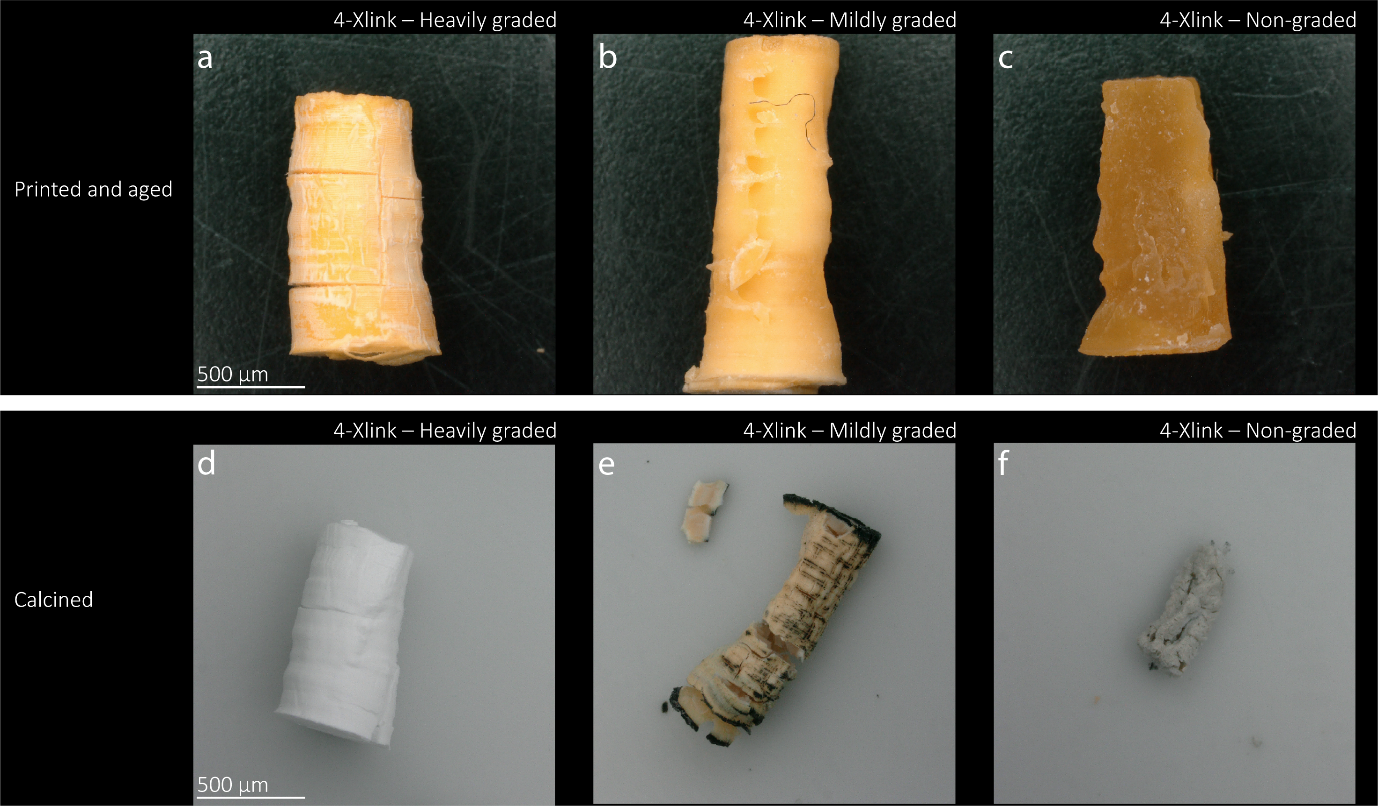


**Figure S1:** Samples printed with resins leading to distinct intralayer morphologies after ageing and after calcination.

**References**

1. Anastasescu, M.; Gartner, M.; Ghita, A.; Predoana, L.; Todan, L.; Zaharescu, M.; Vasiliu, C.; Grigorescu, C.; Negrila, C. Loss of Phosphorous in Silica-Phosphate Sol-Gel Films. J Sol-Gel Sci Technol 2006, 40 (2), 325–333. <https://doi.org/10.1007/s10971-006-8775-y>.
2. Todan, L.; Andronescu, C.; Vuluga, D. M.; Culita, D. C.; Zaharescu, M. Thermal Behavior of Silicophosphate Gels Obtained from Different Precursors. J Therm Anal Calorim 2013, 114 (1), 91–99. <https://doi.org/10.1007/s10973-012-2875-4>.
